# Microstructure, Mechanical and Tribological Properties of Oxide Dispersion Strengthened High-Entropy Alloys

**DOI:** 10.3390/ma10111312

**Published:** 2017-11-15

**Authors:** Xinyu Liu, Hangboce Yin, Yi Xu

**Affiliations:** School of materials Science & Engineering, Southwest Jiaotong University, Chengdu 610031, China; cgvermouth@my.swjtu.edu.cn (X.L.); 17B909079@stu.hit.edu.cn (H.Y.)

**Keywords:** high-entropy alloys, mechanical alloying, metal-matrix composites, Y_2_O_3_ nanoparticles, mechanical properties, tribological properties

## Abstract

A novel metal matrix composite CrMnFeCoNi with Y_2_O_3_ as reinforcement phase was designed and manufactured by mechanical alloying and spark plasma sintering. After sintering at 900 °C for 5 min, the microstructure consisted of a FCC matrix and Y_2_O_3_ nanoparticles. The addition of 0.25 wt % Y_2_O_3_ increased the room temperature tensile strength of the CrMnFeCoNi base from 868 MPa to 1001 MPa, while the mechanical properties of the addition of 0.5 wt % Y_2_O_3_ composite decreased. In the meantime, the addition of Y_2_O_3_ had no significant influence on the coefficient of friction, while the addition of 0.25 wt % Y_2_O_3_ composite shows excellent wear-resistance.

## 1. Introduction

High-entropy alloys (HEAs) have been recognized as the promising candidate for structural applications due to its excellent mechanical properties [1,2]. It usually contains four or five metallic elements with nearly equiatomic ratios. The early studies about HEAs are mainly focused on single crystallographic lattices as simple solid solutions of BCC [3] or FCC [4]. Recent research led to the development of new HEAs, such as hexagonal close-packed (HCP) [5], eutectic mixtures [6,7,8,9], or dual-phase structures of solid solutions [2]. 

CrMnFeCoNi as one of the successful HEAs, has attracted many attentions, especially its mechanical properties in cryogenic temperature [1]. Although, CrMnFeCoNi has a lot of advantages, but its strength is lower in room temperature, so it is imperative to improve its strength. Jae Wung Bae et al. [10] produced CrMnFeCoNi by vacuum induction melting followed by cold rolling and then annealing at for 1 h, the results showed that the yield and tensile strengths were significantly increased, while the elongation did not vary much. Yong Liu et al. [11] prepared CrMnFeCoNi by gas atomization followed by mechanical milling and spark plasma sintering, and the ultimate strength reached up to 1055 MPa, while the elongation was only 6.3%. Joo [12] produced CrMnFeCoNi by high-energy ball milling and spark plasma sintering, and the strength of the alloy increased to 1100 Mpa, but with barely no elongation. Recently, some researchers explore HEA as a matrix for composites with excellent properties. For instance, Rogal et al. [13] produced CrMnFeCoNi with 5 wt % SiC, the composite microstructure consisted of the FCC matrix, SiC particles, M_23_C_6_/M_7_C_3_ carbides and σ-phase. Zhang et al. [14] produced CoCrFeNi self-lubricating composite with graphite and MoS_2_ powders has good mechanical and tribological properties.

In this paper, CrMnFeCoNi HEAs with different content of Y_2_O_3_ were prepared by mechanical alloying (MA) and spark plasma sintering (SPS). The aim was to determine the influence of Y_2_O_3_ nanoparticles on microstructure, mechanical and tribological properties of the new HEA composites and explore their potential for wear-resistant materials.

## 2. Materials and Methods

Three different kinds of Oxide Dispersion Strengthened (ODS) high-entropy alloys were prepared by MA and then sintered by SPS. The high purity elemental powders (purity > 99.5 wt %, particle size < 300 mesh) of Cr, Mn, Fe, Co, Ni were weighted in equiatomic composition and mixed, then the powders were mechanically alloyed in a planetary ball mill (Nanjing Nanda Instrument Co. Ltd., Nanjing, China) for 50 h at 300 rpm. Stainless steel vials and zirconia balls were utilized as the milling media with a ball-to-powder weight ratio of 10:1 under the protection of Ar atmosphere. In order to monitor the alloying process, the powders were extracted at regular at intervals of 0 h, 1 h, 3 h, 5 h, 10 h, 15 h, 20 h, 30 h, 40 h, 50 h. In the meantime, the Y_2_O_3_ nanoparticles (purity ≥ 99.99 wt %, particle size ≤ 30 nm) with mass fractions of 0, 0.25 wt %, 0.5 wt % added in the blend powders at the beginning of the alloying process (the samples are marked as 0-YOHEA, 0.25-YOHEA, 0.5-YOHEA). At last, the as-milled powders were sintered using SPS at 900 °C for 5 min under 50 MPa uniaxial presure with a vacuum of 1 × 10^−3^ Pa.

The crystal structure of as-milled powders and as-sintered bulk HEA composites were examined by X-ray diffractometer (XRD) with CuKα radiation (PANalytical, Almelo, The Netherlands). The morphology and microstructure were observed using scanning electron microscopy (SEM, FEI, Hillsboro, OR, USA) equiped with energy dispersive spectroscopy (EDS, Oxford instruments, Oxford, UK) and transmission electron microscopy (TEM, JEOL, Akishima, Japan), thin foils for the TEM investigations were prepared by the twin-jet polishing in an electrolyte consisting of 20 vol % HClO_4_ and 80 vol % CH_3_OH at subzero temperatures. The density of the bulk HEA compsites was measured by the Archimedes method. The microhardness was measured using Vickers hardness tester (HVS-1000B (Russell Fraser Sales Pty Ltd., Kirrawee, Australia) under the 1 N for 15 s). Tensile properties at room temperature were measured by self-made testing machine and the dog bone type specimens (12 mm × 4 mm × 0.5 mm in size) were measured and the strain rate was 1 × 10^−3^·s^−1^. Sliding wear tests were conducted with a ball-plate contact wear model, using a UMT-2 testing machine (Bruker Corporation, Billerica, MA, USA). The lower plate samples, which are HEA-based composites, and the upper ball, which is GCr15 ball with a diameter of 9.525 mm and a hardness of 766 HV, was moved rectilinearly forwards and backwards. The entire wear process was performed under the following conditions: sliding speed 6 mm/s, moving distance 6 mm, test time 900 s, room temperature (25 ± 2 °C), and one normal load (5 N). After the wear test, the specimens were cleaned with acetone and ethanol and then dried. The wear loss was quantified using a 2D and 3D profiler (NanoMap-D, aep Technology, Santa Clara, CA, USA). The morphologies and tribochemistry of the worn surfaces were analyzed via SEM equipped with energy dispersive spectroscopy (EDS).

## 3. Results and Discussion

### 3.1. Phase Evolution and Microstructure during MA

**Figure 1:**
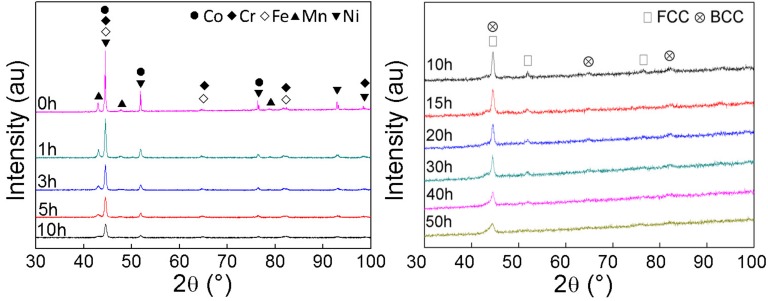
XRD patterns of CrMnFeCoNi powders under different milling times.

As can be seen in the Figure 1, the blend elemental powders show the diffraction patterns of all alloy elements. After milling for 5 h, the pattern of Mn has disappeared at first, suggest that Mn has the highest alloying rate among the five elements, and which can be confirmed by [15]. With the prolonged milling, obvious intensity decrement and peak broadening can be observed, which can be attributed to the grain refinement and high lattice strain [16]. After 50 h of milling, the FCC phase and BCC phase were obtained.

Table 1 shows the crystal size (CS) and lattice strain (LS) of CrMnFeCoNi powders under different milling durations. The CS and LS of the alloy powders under different milling times have been calculated from the X-ray peak broadening using Willison-Hall’s method after eliminating the instrumental contribution. The 50 h milled powders show a CS of 7.97 nm. Meanwhile, the LS of the alloy powders gradually increased from 0.145% to 0.843%. This phenomenon can be attributed to the following reasons: the size difference between different element, severe deformation caused by MA, and the increase in dislocation density [16].

**Figure 2:**
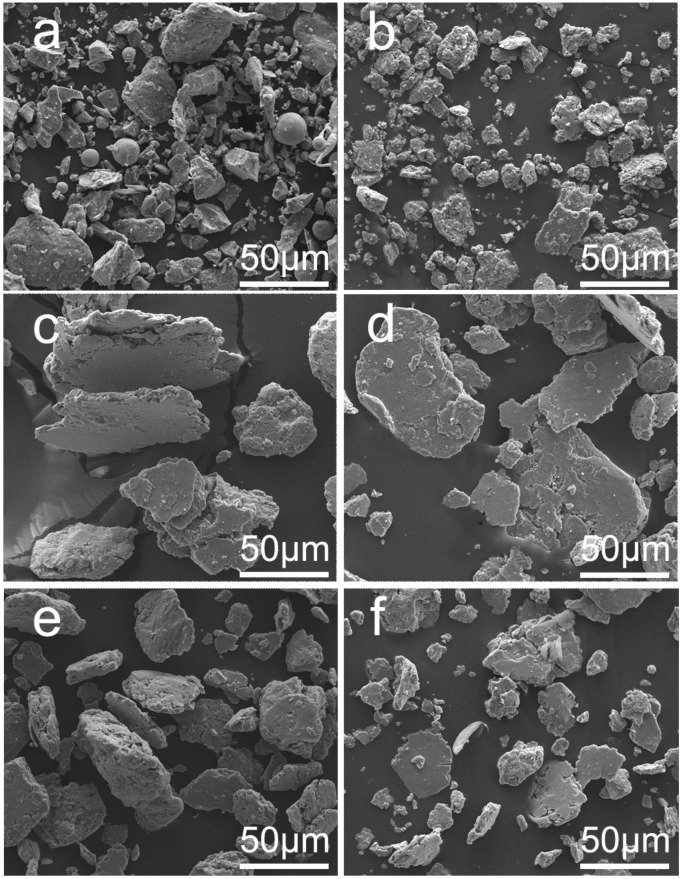
SEM images of CrMnFeCoNi powders with different milling times: (**a**) 1 h; (**b**) 10 h; (**c**) 20 h; (**d**) 30 h; (**e**) 40 h; (**f**) 50 h.

**Figure 3:**
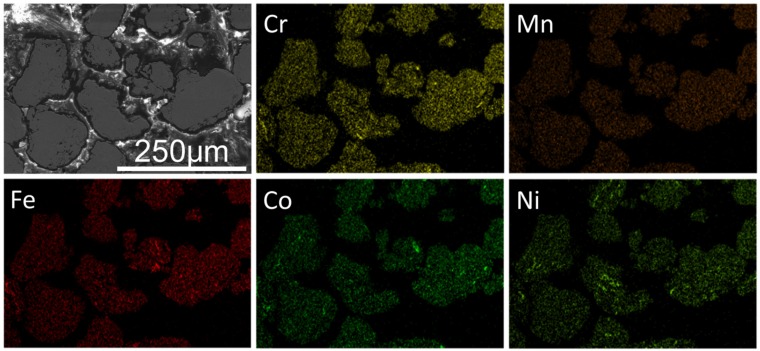
SEM image and EDS maps of 50 h-milled powders.

The second electron images of the CrMnFeCoNi HEA powders of different milling time are presented in Figure 2. During the milling process, flattening, cold welding, fracture, and rewelding occur repeatedly under the action of milling media, and result in severe deformation of the powder particles [17]. The primitive powders exhibited a particle size of less than 20 μm, then the powders are firstly cold welded together and crushed down to a smaller size in the next 10 hours. This process not only promoted the alloying between different elements, but also gradually refined the particles. However, after milling from 20 h to 40 h, the powders were scraped to a lamellar structure while, after milling for 50 h, further refinement had taken place and powders still exhibited a lamellar structure. The EDS analysis results exhibit the excellent chemical homogeneity and the equiatomic composition of alloy particles after 50 h of milling (shown in Figure 3).

**Figure 4:**
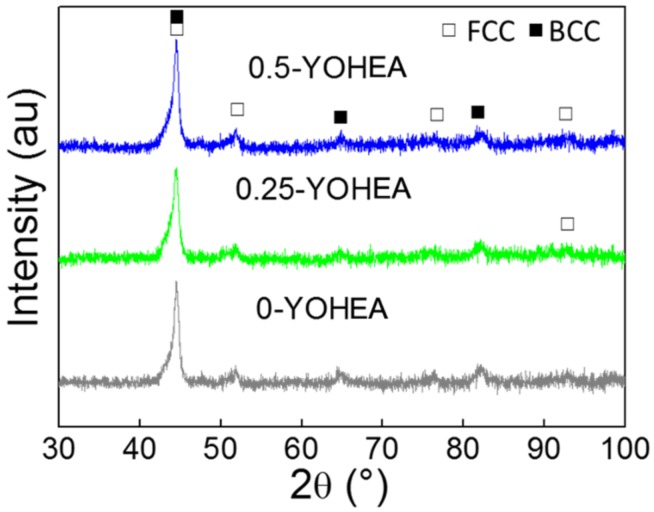
XRD patterns of HEA powders with different Y_2_O_3_ content after milling for 50 h.

**Figure 5:**
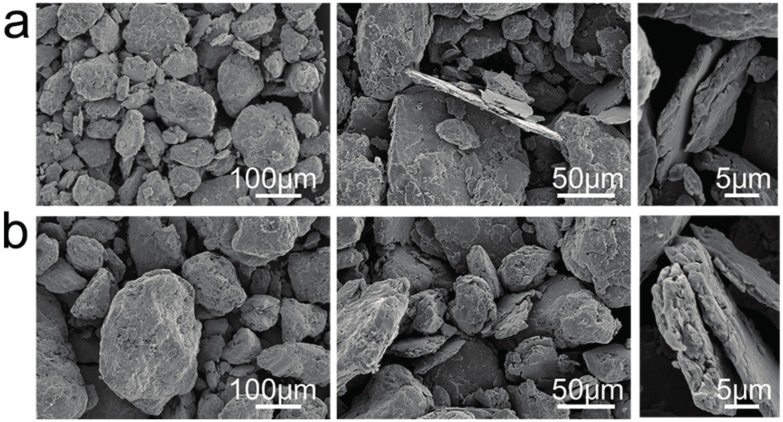
SEM images of HEA powders with different Y_2_O_3_ content after milling for 50 h: (**a**) 0.25-YOHEA; (**b**) 0.5-YOHEA.

Figure 4 shows the XRD patterns of different contents of ODS-HEA powders, compared with the CrMnFeCoNi base powders, no extra peaks can be found in the patterns, which suggest that no new compounds or solid solutions are formed during the milling process. Additionally, the content of Y_2_O_3_ was too low to be detected by XRD. According to Figure 5, the powders exhibit a lamellae morphology, which is caused by the severe deformation of the powders due to the energy generated during the milling process [11,18]. Meanwhile, with the increase of Y_2_O_3_ content, the particle size became larger and larger (0.25-YOHEA: 95.5 ± 24.1 μm, 0.5-YOHEA: 124.3 ± 27.3 μm).

### 3.2. Phase Evolution and Microstructure after SPS

**Figure 6:**
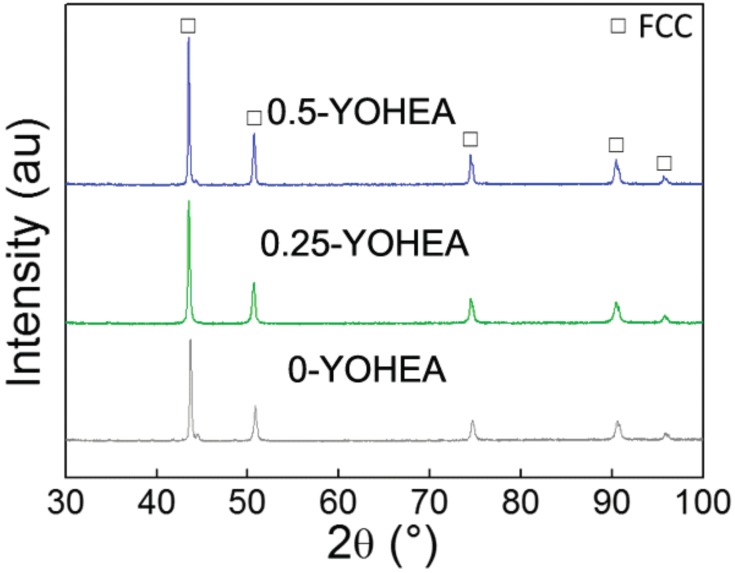
XRD patterns of HEA composites with different Y_2_O_3_ content.

X-ray bulk ananlysis detected occurrence of predominant content of solid solution with FCC structure (Figure 6). There was no difference can be found between CrMnFeCoNi base and ODS-CrMnFeCoNi bulk. Different from [19,20,21], there were no diffraction peaks of carbides be detected in the XRD spectra, indicate that carbon did not react with Cr at sintering. The density of SPSed samples are 7.774 g·cm^−3^, 7.744 g·cm^−3^, 7.727 g·cm^−3^, respectively. Which shows a decreasing trend compared to the theoretical density of CrMnFeCoNi base. No obvious pores can be found in the OM images (not listed in this paper).

**Figure 7:**
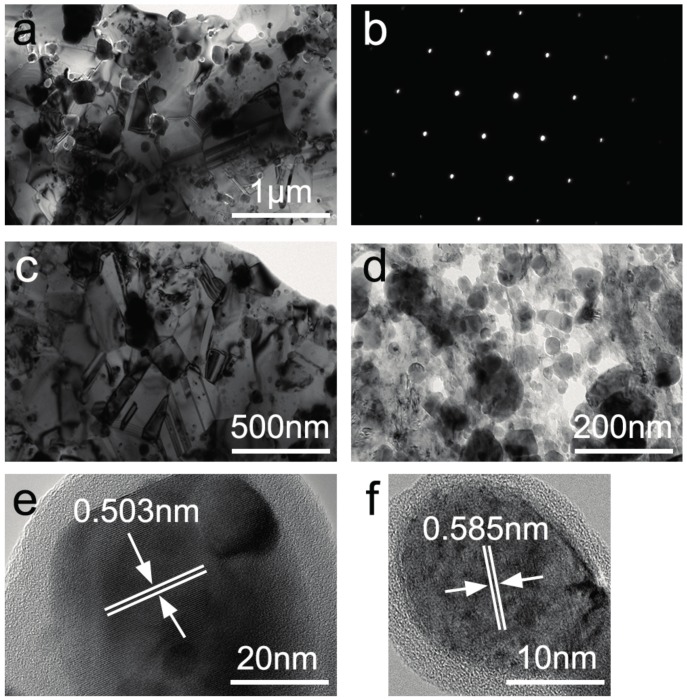
TEM images of SPSed HEAs: (**a**) 0-YOHEA; (**b**) Selected area diffraction in (**a**); (**c**) 0.25-YOHEA; (**d**) 0.5-YOHEA; (**e**) HRTEM image of a single Y_2_O_3_ in (**c**); (**f**) HRTEM image of a single Y_2_O_3_ in (**d**).

TEM and selected area diffraction (SAD) were performed to identify the microstructure of the composites, and the results are shown in Figure 7. The average grain size of three alloys are 210 ± 50.1 nm, 52 ± 23.2 nm, and 31 ± 11.6 nm, respectively. This indicates that the composites have a submicron grain size, which decreases with the increase in Y_2_O_3_ content. It is seen that almost all of the nanoparticles maintain their spherical shapes. Figure 7e–f displays the High Resolution TEM (HRTEM) images of one typical oxide nanoparticles in 0.25-YOHEA and 0.5-YOHEA, which is of approximately spherical shape with a diameter of 40 nm, 20 nm, respectively. The measured interplanar distance of the oxide is 0.503 nm, 0.585 nm, respectively. 

### 3.3. Mechanical Properties

**Figure 8:**
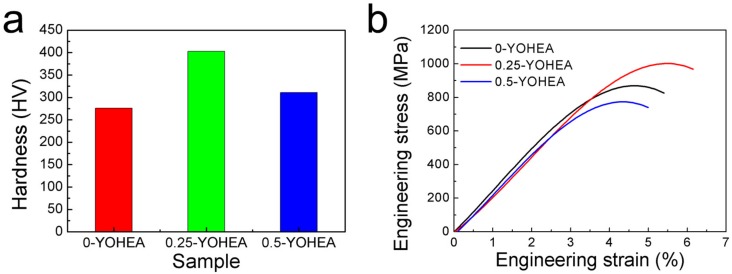
(**a**) Microhardness and (**b**) tensile curves of CrMnFeCoNi composites after SPSed.

Figure 8a exhibits the microhardness of three alloys, and the hardness of each alloy is 276 HV, 403 HV and 311 HV, respectively. According to Hall-Petch relation, the finer the grain size, the higher the hardness while, as can be seen, the HEA containing 0.5-YOHEA has lower hardness. In some materials with nanocrystalline microstructures, softening occurs when the grains are less than 10 to 30 nanometers [22,23,24]. However, there is no good explanation for this phenomenon. 

The results of tensile strain-stress curves of different HEA are shown in Figure 8b and the details are listed in Table 2. It can be clearly seen that the composites with 0.25-YOHEA has the highest tensile strength (TS) and strain. This indicates that the addition of Y_2_O_3_ can significantly improve the tesnsile properties of ODS-HEA composites. The strengthening mechanism of ODS-HEA can be attributed to: (1) Grain boundary strengthening—it is well known that grain size plays an significant role on mechanical properties, which can be explained by Hall-Petch equation; (2) Dispersion strengthening (also known as Orowan strengthening), in which, when dislocation bows bypass the particles, they could lead to the formation of residual dislocation loops and lead to an increase in mechanical properties; (3) The load transfer effect, which relies on the shear transfer of the load from the soft matrix to the hard particles, is a reinforcing mechanism due to a strong cohesion between the matrix and reinforcement [25]. However, the addition of 0.5 wt % Y_2_O_3_ nanoparticles have a negative effect on mechanical properties.

**Figure 9:**
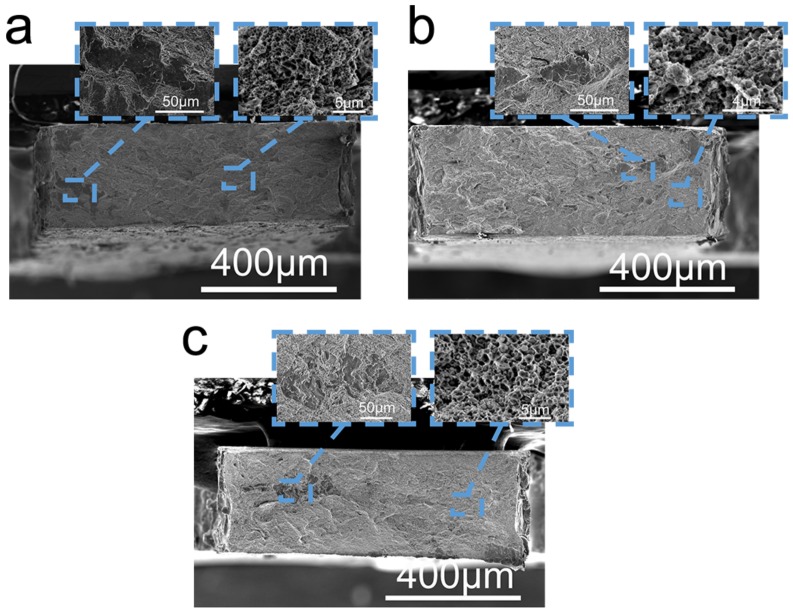
Fracture surface of (**a**) 0-YOHEA, (**b**) 0.25-YOHEA and (**c**) 0.5-YOHEA; the insert shows the cleavage surface and dimples in the corresponding area.

The secondary electron micrographs of fracture images of ODS-HEA composites are shown in Figure 9. Many dimples and a few cleavage surface can be observed, which suggest that the ductile fracture mode is the main fracture mechanism.

### 3.4. Tribological Properties

**Figure 10:**
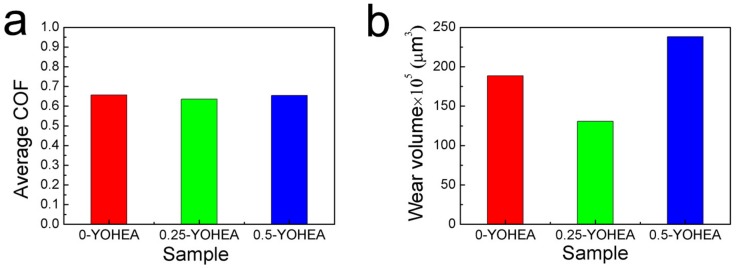
Average COF and wear loss of HEA composites.

The coefficients of friction (COF) and wear loss of composites are shown in Figure 10. It can be seen that the addition of Y_2_O_3_ has made no difference on the COF, the 0.5-YOHEA has the highest wear loss under the same load, and the 0.25-YOHEA is the lowest one. Compared with other reported composites [26,27,28], the ODS-HEA composites always had excellent anti-wear and friction-reducing abilities. 

**Figure 11:**
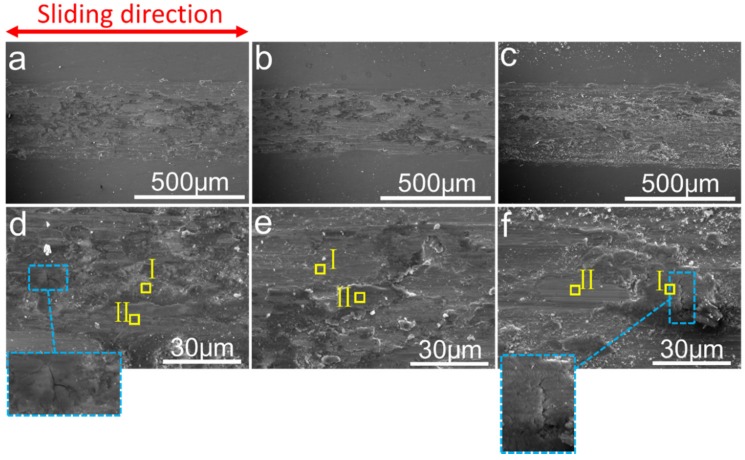
SEM morphologies of the worn surface of the HEA composites (**a**), 0-YOHEA (**d**), 0.25-YOHEA (**b**,**e**) and 0.5-YOHEA (**c**,**f**).

For the purpose of investigating the wear mechanism, SEM and EDX were performed to analyze the worn surfaces of composites, as shown in Figure 11. Under the load of 5 N, Figure 11a,d show the worn surface of 0-YOHEA composite has some furrows on the surface of the abrasive, a small amount of debris, a black adhesive, a fold deformation, and microcracks perpendicular to the direction of the friction movement, The worn surface of 0.25-YOHEA has furrows, a small amount of debris, black adhesion and perpendicular to the direction of friction movement of the fold deformation (Figure 11b,e), while the worn surface of 0.5-YOHEA has deeper furrows, a lot of debris, black adhesion, perpendicular to the direction of friction movement of the fold deformation, and micro-cracks (Figure 11c,f). The results indicate that the wear ratio increases with the increase of the content of Y_2_O_3_ nanoparticles. The Y_2_O_3_ as a kind of abrasive particles, possibly applied to friction and wear interface.

The EDS results (Table 3) shown that the abrasive black adhesion and wrinkled area (marked with I in Figure 11d–f) had higher oxygen content than the other regions (marked with II in Figure 11d–f). The material surface produces an oxide layer that prevents direct contact between the steel ball and the alloy, resulting in a lower friction shear strength and a lower adhesive strength in the friction contact area, which can be reduced by reattaching the abrasive particles of the loss, the oxide layer has the role of solid lubricants to reduce friction and wear on the grinding system [29,30]. Above all, the main wear mechanisms were abrasive wear, adhesive wear, and oxidation wear. 

## 4. Conclusions

The microstructural, mechanical, and tribological properties of oxide dispersion strengthened CrMnFeCoNi high-entropy alloys composites were investigated. Based on the analysis and tests, the following conclusions are drawn.

1. A new ODS-CrMnFeCoNi HEA matrix composite was successfully prepared from elemental powders by using MA and SPS. The as-milled HEA powders exhibited FCC phase and BCC phase. After sintering, the composite material consists of a single FCC structure and Y_2_O_3_ nanoparticles in homogeneous dispersion state.
2. With increasing content of Y_2_O_3_, only 0.25 wt % Y_2_O_3_ nanoparticles can improve the strength to 1000 MPa, which was 15.2% higher than the CrMnFeCoNi matrix. Additionally, the strain also increased from 5.4% to 6.1%. This is mainly because of the grain boundary strengthening effect, orowan looping, and load transfer effect. However, with 0.5 wt % Y_2_O_3_ nanoparticles, both the strength and strain were decreased, and this may contributed to some complicated reasons.
3. The addition of Y_2_O_3_ nanoparticles increased the hardness of alloy, from 246 HV to 403 HV and 311 HV, respectively. The increase in hardness due to the fine grain strengthening effect, while the decrease in hardness may be caused by the inverse Hall-Petch effect.
4. All of the composites exhibited a similar COF, which showed the excellent friction-reducing abilities, meanwhile the addition of 0.25 wt % Y_2_O_3_ nanoparticles showed the best anti-wear abilities.

## Figures and Tables

**Table 1:** crystal size (CS) and lattice strain (LS) of HEA powders under different milling durations.

Milling Time (h)	CS (nm)	LS (%)
1	30.35414	0.145
3	23.81794	0.178
5	21.61512	0.205
10	17.23008	0.245
15	14.33161	0.307
20	12.83324	0.386
30	11.14773	0.468
40	10.17895	0.575
50	7.972322	0.843

**Table 2:** Details of mechanical properties of CrMnFeCoNi composites.

Sample	TS (MPa)	Strain (%)	Microhardness (HV)
0-YOHEA	868	5.4	276
0.25-YOHEA	1000	6.1	403
0.5-YOHEA	773	4.9	311

**Table 3:** EDS results of HEA composites marked in Figure 11.

Sample	Place of Analysis	Element (at %)							
		Cr	Mn	Fe	Co	Ni	O	Y	C
0-YOHEA	I	7.59	7.37	14.98	7.27	7.13	55.66	-	-
	II	15.61	15.18	15.17	14.3	13.6	8.97	-	17.17
0.25-YOHEA	I	8.45	8.32	15.62	7.7	7.43	52.1	0.16	-
	II	14.33	15.1	16.86	12.34	14.52	6.41	0.1	20.06
0.5-YOHEA	I	9.56	9.64	13.13	9.35	8.9	35.1	0.04	14
	II	19.06	18.63	21.05	17.92	17.72	4.74	0.43	-
